# Cancers of unknown primary origin: current perspectives and future therapeutic strategies

**DOI:** 10.1186/1479-5876-10-12

**Published:** 2012-01-24

**Authors:** Giulia Maria Stella, Rebecca Senetta, Adele Cassenti, Margherita Ronco, Paola Cassoni

**Affiliations:** 1 Department of Oncological Sciences, Institute for Cancer Research and Treatment (IRCC), 10060 Candiolo (Turin), Italy; 2 Department of Molecular Medicine, - Section of Pneumology, Laboratory of Biochemistry & Genetics; University and Fondazione IRCCS Policlinico San Matteo, 27100 Pavia-Italy; 3 Department of Biomedical Sciences and Human Oncology, University of Turin, 10128 Turin, Italy

**Keywords:** Cancer, Metastases, Invasive growth, Mutations

## Abstract

It is widely accepted that systemic neoplastic spread is a late event in tumour progression. However, sometimes, rapidly invasive cancers are diagnosed because of appearance of metastatic lesions in absence of a clearly detectable primary mass. This kind of disease is referred to as cancer of unknown primary (CUP) origin and accounts for 3-5% of all cancer diagnosis. There is poor consensus on the extent of diagnostic and pathologic evaluations required for these enigmatic cases which still lack effective treatment. Although technology to predict the primary tumour site of origin is improving rapidly, the key issue is concerning the biology which drives early occult metastatic spreading. This review provides the state of the art about clinical and therapeutic management of this malignant syndrome; main interest is addressed to the most recent improvements in CUP molecular biology and pathology, which will lead to successful tailored therapeutic options.

## Introduction

Cancer of unknown primary (CUP) origin defines metastatic tumour detected when the site of primary origin cannot be identified based on clinical history, complete physical examination, routine laboratory tests, imaging and radio-metabolic techniques and careful review of histological specimens. Although this malignant syndrome accounts for 3-5% of all cancer diagnosis, the majority of patients still lacks effective therapeutic regimens [1] CUP clinical presentation is extremely heterogeneous: about 15-20% of CUP patients can be assigned to favourable subsets whereas the others share a very aggressive potential and unpredictable pattern of metastatic spread. The largest group of these tumours is refractory to standard chemotherapy and the median survival of CUP patients is very low.

There is poor consensus on the extent of diagnostic evaluations in front of metastatic cancers without a primary mass. At the present immunohistochemistry (IHC) is often the only standard method by which a putative primary origin can be postulated. Nevertheless, recent improvements in molecular diagnostics will allow a most appropriate pathological classification of early metastases. However the most relevant approach to CUP is related to the understanding of the biology which drives precocious metastatic spreading.

This review summarizes the current knowledge on pathological features, diagnostic tools and biological behaviour of CUPs, mainly focusing on the next future therapeutic implications.

## CUP: definition, epidemiology and clinical approach

Almost one third of advanced tumours presents with metastases at time of diagnosis. In the majority of cases the organ site of primary lesion becomes shortly evident after clinical, pathological and radio-metabolic evaluations. The other cases can be roughly defined as metastases of unknown primary origin. Indeed this definition includes two groups of tumours: 1) the cases in which the primary site might be postulated at least by their ICH profile; 2) the metastatic lesions which remain really 'orphan' even after an exhaustive IHC screening. Although the separation into two groups based on the IHC profile could be somehow artificial, it may be important for patient management and the choice of initial therapeutic regimen.

It is generally reported that the incidence of CUPs is about 3-5% of all cancer diagnosis [2]. The annual age-adjusted incidence per 100,000 population in USA is 7-12 cases; in Australia 18-19 cases and in the Netherlands 5.3-6.7 cases [3]; however it must be kept in consideration that the incidence might be higher since some CUPs patients are classified for pragmatic clinical reasons as 'known primary' even if their diagnosis is uncertain. The median age at diagnosis is reported to be 60 years and the occurrence is slightly higher in males. In some instances, although the metastatic pattern is often unpredictable, the site of primary origin can be found during lifetime or autopsy: it is generally a small nodule often localized in the lungs or in the bilio-pancreatic tract [4].

In the early 1970s some researchers argued that diagnosis of cancer of unknown primary origin could only be made if the primary tumour was not found at autopsy [5]. At the present, as suggested by International Guidelines (http://www.nccn.org,http://www.esmo.org), all the patients who present with a metastatic cancer suspected to be of unknown primary should have an accurate physical examination, complete laboratory tests and a whole body imaging study (Computed Tomography - CT - scan and Positron Emission Tomography - PET). Besides, female patients have to undergo mammography and vaginal ultrasound (US) scan, whereas prostate US scan is required in males. Various endoscopic procedures (laryngoscopy, bronchoscopy, gastroscopy, colonoscopy or cystoscopy) should be ordered in selective cases, based on several clinical factors such as patients' relevant symptoms and/or signs, physical examination findings, sites of metastases, occult blood in the stool, laboratory findings as well as any other factor which would prompt endoscopies. More than 50% of CUP patients present with multiple sites of involvement while the rest have a single site, most commonly liver, bone, lung or lymph nodes [6].

CUP patients are classified into subgroups and specific risk categories according to the organs involved (disease stage) and histology in order to optimize patient management [7]. A minority (15-20%) of patients belongs to the subset at more favourable prognosis: these patients harbour the most differentiated and chemosensitive tumors and have the longest survival rates. Unfortunately the majority of cases do not belong to any specific category. These cases have the worst prognosis, displaying a substantial resistance to therapy. Unfavourable predictor factors are related to diagnosis of: adenocarcinoma metastatic to liver, non-papillary malignant ascites, multiple cerebral metastases, multiple lung/pleural metastases, systemic bone disease. Better prognosis is, on the other hand, related to: poorly differentiated carcinoma with midline-distribution, papillary adenocarcinoma of peritoneal cavity (in women), adenocarcinoma involving only axillary lymph nodes (in women), squamous cell carcinoma involving cervical lymph nodes, isolated malignant adenopathy, poorly differentiated neuro-endocrine carcinoma, single small and potentially resectable tumour [2,6]. Notably, it seems that CUP survivors have a higher risk of developing many subsequent cancers [8]. The overall prognosis of CUP patients is generally very poor with a median survival of 4-12 months, with about 50% of patients alive at 1 year and about 10% at 5 years from diagnosis [9]. Therapy is currently designed on the bases of clinical-pathological investigations and according to disease staging and risk assessment; at the present the optimal chemotherapeutic regimens remains to be clarified and the most commonly used regimens contain platinum [1].

## Pathologic presentation and analysis

**Figure 1:**
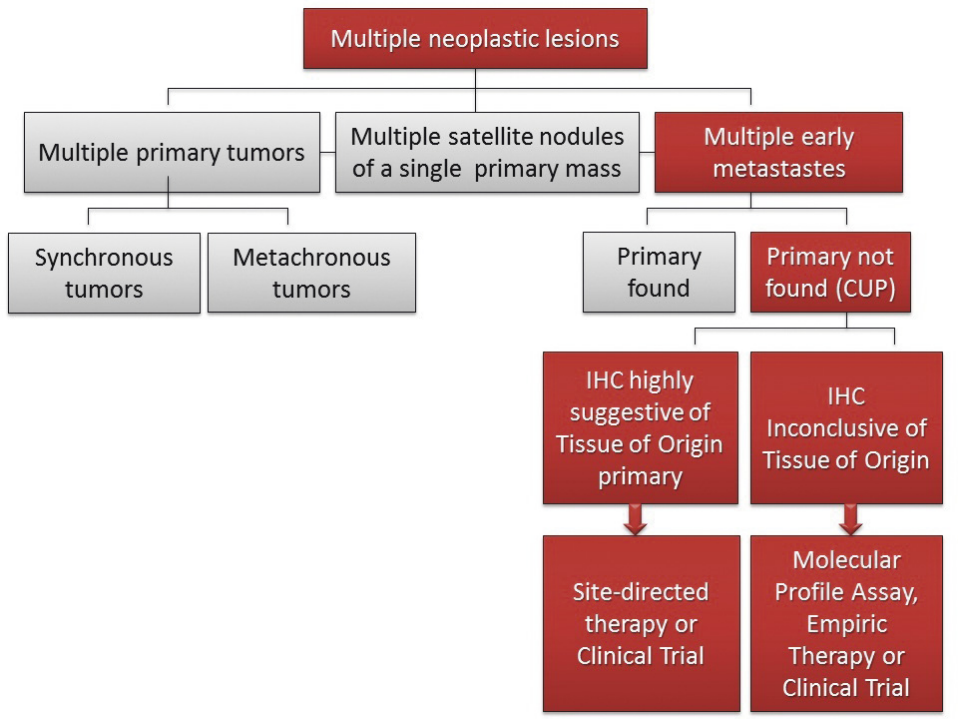
**Tacking the primary: from multiple malignant nodules to CUPs**.

Cancer can arise in any tissue of the body. In the vast majority of cases, the first step in cancer onset is the growth of a primary lesion; only later a metastatic clone acquires the biological properties required to detach from the mass, invade lymphatic or blood vessels and eventually colonize distant organs. Nevertheless some exceptions from this behaviour might be underlined. Sometimes tumours arise as multiple primary lesions. They could be: i) synchronous tumours and can display different histology or share the same histology but be anatomically separated; ii) metachronous nodules with the same histology, but temporarily separated each other. Multiple nodules can also identify satellite nodules of a primary lesion if they share the histology of the primary tumor and are spatially separated within the same organ. Finally multiple neoplastic nodules might be metastases, which have spread from a primary tumor and grown with a spatial and temporal (< 2 years) interval from it. From the anatomic perspective, metastasis can leave the primary site through a cavity (e.g. from lungs to the pleural cavity or from colon to peritoneum); by lymphatic spreading or through blood vessels. Cancer cells can even migrate across the endothelium, the basement membrane or along neurons [10-13]. In general metastatic cells maintain the histologic features of the tissue from which they derive, even in case of upfront metastatic cancers. However, in a number of cases, cancer cells appear as multiple nodules which do not display morphology and properties referable to the tissue/organ in which they are arising or cannot be related to any suspected distant primary site. The latter identify cancers of unknown primary (CUP) origin (Figure 1).

When CUP is suspected, the diagnostic algorithm followed by pathologists has to be--at the same time--strict and extensive. The vast majority of CUPs are represented by carcinomas; with respect to the cancer morphology, CUP can be classified as: i) well, moderately or poorly differentiated adenocarcinomas; ii) squamous cell carcinomas; iii) poorly differentiated carcinomas, iv) carcinomas with neuroendocrine differentiation; v) undifferentiated cancers. Adenocarcinomas of unknown primary are the most frequently diagnosed. In such settings, immunohistochemistry is the only standard test which could be the determining factor to suggest the primary origin of the lesion. Several immunohistochemical markers have been proposed to predict the site of the primary tumor. As recently suggested by Greco FA et al. [14] the screening panel might include citokeratins (CK-7; CK-20), TTF-1; breast/ovarian markers, HEPAR-1, renal cell, placental alkaline phosphatase/OCT-4, WT-1/PAX8, synaptophisin and chromogranin. IHC accurately predicts a (single) primary in ~ 35/40% of early metastatic cancers [6]. It has been demonstrated that panels of markers are superior to single biomarkers in identifying the primary site, at least in adenocarcinomas. Dennis JL and coll. [15] provided correct primary site identification in 88% of cases of metastatic adenocarcinoma applying an immunohistochemical diagnostic algorithm including 10 immunohistochemical markers. However it should be noted that these studies were done in patients with known metastatic cancers, rather than in patients with CUP. Thus, their findings, although important, may not be entirely analogous to patients with unknown primary cancer. Besides, the pathologic evaluation is in the vast majority of cases performed on formalin-fixed paraffin-embedded (FFPE) small sized samples, mainly derived from bioptic procedures. Besides, the choice of IHC panel deeply influences subsequent diagnosis [16,17]. Moreover IHC lacks of specificity and sensitivity in staining some primary tumors, such as upper gastrointestinal cancers (gastro-esophageal and pancreatico-biliary tumor). As a consequence, in a number of cases the IHC markers profile may only suggest a differential diagnosis rather than indicate a conclusive single diagnosis.

**Figure 2:**
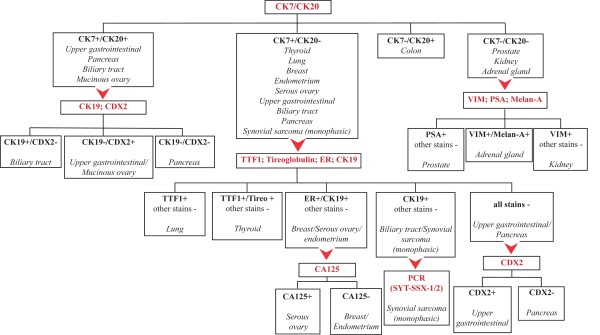
**Diagnostic algorithm to characterize metastatic adenocarcinoma from unknown primary**. (CK: cytokeratin; TTF1: thyroid transcription factor 1; ER: estrogen receptor; CA125: cancer antigen 125; tireo: tireoglobulin; VIM: vimentin; PSA: prostate specific antigen)

In conclusion, the diagnostic procedure begins if necessary by determining the cell lineage (epithelial, melanocytic, lymphoid, mesenchymal, germinal cells) with the aid of appropriate markers; the IHC panel will be then determined coherently. Here we propose a hypothetical ideal IHC diagnostic algorithm for screening and issuing towards tumor primary, which may be used once the tumor has been confirmed to be a carcinoma (Figure 2). It should be remarked that, although complete and concise, realistically, this approach could not be recommended in all patients. The clinical circumstances, including the patient's gender, site of metastasis, histologic appearance of their tumor and the screening immunohistochemistry of CK-7, CK-20, TTF-1 and CDX-2 might be considered as first step and subsequent staining needs to be individualized, based upon patient' s clinical pathologic setting.

There are few reports focusing on the validation of the predictive value of the immunophenotype in CUPs. From this perspective, classical pathologic approach can be integrated by molecular biology and gene expression profiling studies. In 2008 Horling H and coll. [18] showed that the IHC prediction of primary was consistent with the molecular profiling in a series of adenocarcinoma of unknown primary.

In summary, in some cases IHC algorithms can allow the identification of a primary site with adequate accuracy but, at the present, there are not enough precise knowledge to definitely distinguish two different groups, those with and without IHC-solved primary. As a consequence, unless patients have a clinically defined and anatomically recognized primary site--discovered at the time of the workup or later in the course of their disease, have CUP. It means that--at the present--there is no enough precise knowledge to state that those cancers without a strong IHC single diagnosis are definitely different from the others. However, a precise and recognized immunohistochemical profile may lead to appropriate treatment in individual patient's settings. This approach has been clearly supported by Varadhachary GR and coll. [19] and more recently by Greco FA and Hainsworth JD [20]: in both cases, authors described a CUP featuring the CDX-2 and CK20 +, CK-7 staining profile and they defined it as a '*colon cancer profile CUP (CCP-CUP)*'. Importantly, it has been shown that this subset of patients better responds and seems to have a superior outcome when treated with site-specific therapy.

## The role and contribution of imaging

Detection of the primary tumor may optimize treatment planning, which, in turn, may improve patient prognosis. This goal might be considered in front of a suspect of metastatic cancers of occult primary. Since the unknown primary mass can be located anywhere in the body, a cross-sectional whole-body imaging modality is the proper method to look for a primary site. Ultrasound is a fairly quick and easy procedure that doesn't use radiation, which is why it is often one of the first tests done if an internal mass is suspected; computed tomography (CT) and magnetic resonance (MRI) thus represent the imaging studies most used in clinical practice to dissect the whole body. The combination of 18 F-fluorodeoxy-glucose (FDG) positron emission tomography (FDG-PET) and CT has gained wide acceptance, especially if the primary tumor is unknown. It should be noted that small lesions or pathological changes in normal-sized tissues can be missed by CT and MRI; this is especially relevant in CUP setting in which the primary tumor is supposed to be a small lesion [21]. From this perspective, positron emission tomography using the radiotracer 18 F-fluoro-2-deoxyglucose, is the leading approach since it provides functional and metabolic information with an excellent lesion *vs *background ratio. Notably, studies of unknown primary malignancies are among the most appropriate indications for PET, according to international nuclear medicine guidelines [22]. Integration of PET/CT scanner (equipped with a 16- to 64-section multidetector-row CT unit) is rapidly replacing the PET study alone and is at the present the preferred method of choice for the detection of primary tumors in patients with CUP. Notably, PET/CT imaging is known to have a good sensitivity and specificity, mainly in head and neck and lung cancers [23]. A true positive result is considered when the imaging-suggested putative primary is subsequently confirmed through biopsy. On the other hand, a false negative result is considered if the primary tumor is detected in a site that is negative on the imaging technique. A recent meta-analysis [24] showed that, overall, FDG-PET/CT is able to detect 37% of primary tumors in patients with CUP, with both sensitivity and specificity of 84%. Although data as a whole are encouraging it should be noted that cohorts of CUP patients analyzed in each study are extremely mixed and heterogeneous (Table 1).

**Table 1:** Predictive values of PET in tracking the primary site in case of early metastatic cancers

Match PET/histology in identification of the primary lesion	Total examined cases	Predictive value of PET% (n of patients)	False positive cases% (n of patients)	Reference
Failed	1	--	---	[25]

Partial	23	56,5 (13)	---	[26]

Partial	39	27 (6)	2.5 (1)	[27]

Failed	18	--	---	[28]

Partial	20	11(55)	5 (1)	[29]

Partial	149	24.8 (37)	8.7 (13)	[30]

Failed	20	--	---	[31]

Partial	51	9.6 (5)	---	[32]

Partial	77	36.4 (28)	1.2(1)	[33]

Partial	24	37.5 (9)	12.5 (3)	[34]

Complete	1	1	---	[35]

Partial	59	59 (35)	---	[36]

Partial	44	31.8 (14)	11.3 (1)	[37]

Partial	47	14.8 (7)	27.6(13)	[38]

Partial	13	7 (54)	---	[39]

Partial	43	55.8 (24)	2.3 (1)	[40]

Partial	67	53.7 (36)	1. 49 (1)	[41]

Partial	430	31.4 (135)	3.9 (17)	[42]

Partial	60	30 (18)	21.6 (13)	[43]

Partial	39	26 (10)	---	[44]

Partial	15	20 (4)	6.6 (1)	[45]

Partial	14	40 (7)	7.1 (1)	[46]

Partial	38	53 (20)	2.6 (1)	[47]

In conclusion the diagnostic challenge for PET/CT is to minimize the false negative in tracking the primary tumor. FDG is a nonspecific radiotracer with can accumulate also in no-malignant areas of increased glycolysis, such as inflammatory areas. A number of radiotracers are under investigation including compounds that can mark hypoxia, angiogenesis and apoptosis in tumors. Advances in PET technology and the integration of PET/MRI as well as improvements of the hardware for data analysis are expected to improve the management of CUP patients.

## Molecular profiling for the identification of the primary tissue-of-origin

Despite the large number of patients diagnosed with carcinoma of unknown primary site of origin, innovative and individualized approaches to managing these patients have lagged behind many other solid tumors. At the present great efforts are directed to take advantages from the new microarrays technology to predict the tissue-of-origin (ToO) of CUP. This approach is eventually driven by the hypothesis that the knowledge of the putative primary could help customizing therapy and thereby improve clinical outcome.

Several methods for identifying CUP samples based on their gene expression profiles have been developed. Talantov D and coll. [48] and Varadhachary GR and coll. [49] have presented an RT-PCR based method that measures the expression of 10 signature genes. Ma XJ and coll. [50] proposed a similar method based on 92 genes, which resulted in an overall accuracy of 82% among 39 cancer types. Tothill RW and coll. [51] presented a support vector machine based method for classifying cancer types, and selected 79 genes for an RT-PCR test reaching a total accuracy of 89% but only among 13 cancer types. Rosenfeld N and coll. [52] applied a similar approach, but instead of measuring traditional gene expression, they looked at miRNA expression to classify CUP samples. For a majority of the samples, they achieved a ~90% classification accuracy.

There are currently several commercial tests available with gene expression-based assays that classify tumors of unknown or uncertain origin: Pathwork [microarray for messenger RNA (mRNA)], Rosetta Genomics and Prometheus (RT-PCR for microRNA), and bioTheranostics (RT-PCR for mRNA). All tests claim prediction accuracies in known primary cancers between 80% and 90% [14]. However, each test has different specimen requirements and a wide range in their ability to identify cancer types/subtypes (15-54 types/subtypes). In general, a clinically viable test needs to be compatible with FFPE tissues with low numbers of tumor cells and to have the ability to discriminate large number of tumor types since metastatic cancers can arise from many cancer types and primary tumor sites. More recently Varadhachary GR and coll. [53] showed in a perspective study that ToO can be identified by using a microRNA-based assay in a high percentage (84%) of metastatic cancers. Authors' conclusion suggested that microRNA assay may be most helpful in guiding management when IHC studies are unconclusive or provides a large differential diagnosis. Therefore, where applicable, those tests may be intended as complement to IHC to provide an multidisciplinary network for a most appropriate clinical management of CUP patients. Similar conclusions have been reached by Ferracin M and coll. [54] that demonstrated that FFPE samples can be used to reveal the ToO of metastatic cancer by using miRNA expression profile and suggested that this approach could provide useful indications for CUPs. It has also been documented that Pathwork test can be adequately performed on FFPE cell blocks from cytologic body fluid specimens material and can be used in the identification of ToO in case of metastases of unknown primary [55]. In all the reported works, authors argue that the availability of ToO holds promise for the increasing individualization of therapy for CUP patients [20]. Authors' ultimate goal is to provide a helpful framework where profiling and pathology are integrated in a cost and clinically effective algorithm with a positive impact on patient survival and quality of life. Molecular profiling for the identification of the ToO is a promising technique to improve the site of origin diagnosis in CUP patients, which could be exploited in the clinical practice. Interestingly, prospective clinical trials are ongoing to determine whether treatment based on molecular profiling can improve CUP patient outcomes. A major issue is to determine if CUP patients respond similarly to the corresponding subsets of patients with known primary when treated with site-specific therapy, based upon IHC profile and/or molecular profiling assay. Besides, gene expression assays will be promising for diagnosis and hence therapy, in those cases displaying inconclusive IHC profile.

## Current therapeutic approach to CUPs

Surgical management of metastases of unknown primary is generally related to limited/single disease. Nevertheless, a recent report demonstrated for the first time that surgery in case of multiple sites of spinal disease did not influence survival; whereas the presence of extraspinal disease had a negative impact [56]. However since CUP syndrome mainly presents with advanced disease systemic therapy is the most frequent approach.

The continuing development of new therapies targeted to the various cancer types makes mandatory the identification of the primary tumor and--as discussed above--many efforts are now directed to integrate molecular based medicine and clinical practice to assign a primary as soon as possible after diagnosis of metastases of occult primary origin. However in case of CUP diagnosis, therapy regimes lack to be specific and, in the vast majority of cases, chemotherapy regimens include platinum, an alkylating agent which is effectively used for the management of patients with the most common solid tumors. In those settings, treatment may only help to control metastatic disease for a time and to improve patient symptoms. Overall clinical prognostic markers include: patient's age, gender, weight loss and performance status; histopatology and tumor burden, location and number of metastatic sites; as well as serum biomarkers [2]. In addition, the French CUP Group (GEFCAPI) developed a simple prognostic index for patients with CUP, in which favorable prognostic factors included a performance status < 2 and normal serum LDH levels [57]. Several other algorithms are under investigation, with the aim to help clinicians involved in CUP patients care. Interestingly, the survival of CUP patients who are enrolled in clinical trials significantly higher (6-10 months) if compared to that of unselected CUP patients who are not enrolled in clinical trials (2-3 months) [58].

For adequate therapeutic guidance CUP entities should be categorized into favorable or unfavorable subsets [59]. In the recently published ESMO guidelines [1], it has been clearly stated that treatment has to be tailored on an individual basis according to the clinical-pathological subset of distinct prognosis in which the patient belongs. About 10-15% of CUP patients of the favorable risk subsets should be treated similarly to patients with equivalent known primary tumors with metastatic dissemination. As discussed above, patients who belong to these subcategories have a better prognosis and undergo specific recommended treatments. Indeed in those cases, retrospective analyses have shown that patients'outcome displays no substantial differences from those with metastatic tumors of known primary. On the other hand, patients with poor-risk CUP have a dismal prognosis despite management with a variety of chemotherapeutic combinations in small clinical studies. Notably, no superior efficacy has been proven--till now--with respect to any of the tested schedules incorporating platinum, taxanes or third generation compounds [60].

Carboplatin plus paclitaxel combination chemotherapy has been reported to be effective in patients with predominantly nodal/pleural metastases of unknown primary carcinoma and in women with peritoneal carcinomatosis but showed lower benefits in patients affected by liver, bone or multi-metastatic disease [61]. Similar results were obtained by Park YH and coll. in 2004 [62] whose study evaluated the efficacy and toxicity of combined paclitaxel and cisplatin chemotherapy in a subset of CUP patients, mainly affected by adenocarcinomas. In this phase II study the median survival was 11 months whereas the median time to progression was 4 months. In 2007 Pentheroudakis G and coll. published a study demonstrating that combination docetaxel carboplatin is a safe and effective palliative option for CUP patients [63]. Also combinatorial triplets with gemcitabine or etoposide, carboplatin, and paclitaxel seem to be tolerable treatment for CUP patients [64,65]. A recently closed phase II trial evaluated the efficacy and toxicity of the combination of paclitaxel, carboplatin, bevacizumab, and erlotinib in the first-line treatment of CUP patients [66]. All the enrolled patients received the four drugs and treatment cycles were repeated every 21 days. When carboplatin/paclitaxel were discontinued, bevacizumab/erlotinib were continued until tumor progression. This empiric drugs combination allowed a median progression-free survival time of 8 months, with 38% of patients being progression free at 1 year. The median survival time and 2-year overall survival rates were 12.6 months and 27%, respectively; toxicity profile was well tolerated. Randomized prospective trials proving that any form of chemotherapy improves the survival of CUP patients with disseminated adeno--or undifferentiated carcinoma over best supportive care alone are still lacking. Nevertheless platinum-based combination schedules represent the first line approach to CUP treatment and because of the lower toxicity of carboplatin as compared to cisplatin, the favored regimen is carboplatin/paclitaxel. With this combination, response rates of 30-40% and a 2-year survival rate of 20-25% can be achieved if administered as first-line therapy [67]. Few data are available about second-line chemotherapy in CUP patients who already received platinum-based first-line treatment. Although it remains unclear whether second-line chemotherapy might contribute to a survival benefit in patients with CUP, patients who show a favorable response to first-line chemotherapy appear to be likely to benefit from second-line chemotherapy [68]. Chemotherapy regimens evaluated include the combination of oxaliplatin and capecitabine which has been found to have activity as salvage treatment for patients with CUP, mainly in those feature a so called 'colon cancer profile-CUP' [69].

Radiotherapy is a conventional therapeutic approach in case of cervical lymphnodes metastases of unknown origin, not only for unresectable disease but for a comprehensive treatment of the whole neck. Indeed neck dissection is not always necessary; for instance, it is not necessary for N1 disease [70]. Intensity modulated radiation therapy (IMRT) can produce excellent outcomes without relevant long term complications [71]. Definitive IMRT to 50-56 Gy followed by neck dissection seems to result in excellent nodal control and overall and disease-free survival, with acceptable toxicity for patients with localized non-bulky disease without extracapsular spread, T0N1 or non-bulky T0N2a stages [72]. Relapse occurred infrequently in patients treated with excisional biopsies and postoperative radiotherapy. These results appear consistent with those expected for patients with advanced neck disease and a known primary site [73]. In summary results from chemotherapy and radiotherapy on CUP patients do not exclude them from studies aiming to improve their outcomes. Indeed, in the last decade it has been largely shown that cancers belonging to the same tissue/organ do not respond to treatment in the same way, and response to treatment lies inside their genome: presence or absence of specific genetic lesions (either mutations or amplifications) can really predict responses to therapy. Ultimately a mutational profile, rather than prediction of ToO, could be useful to treat patients with targeted therapies. Steady collaboration and fluid communication between oncologists, pathologists and molecular biologists is a clear priority for the correct interpretation of tests and the personalized approach required by each individual CUP case. Work in multidisciplinary teams will result in significant changes in the diagnosis and treatment of these patients

## CUP: unknown primary or unknown biology?

Metastatic spread is generally considered the final step of tumor progression and it clinically sets up the worse level of the cancer staging system. According to this model, the early cancer diagnosis is the one able to detect a single localized tumor mass which is still susceptible of surgical exeresis. However, as discussed above, in 3-5% of all human cancers distant dissemination arises at an early stage of tumor progression, so that unexpected metastatic phenotype can overcome on the primary lesion's growth. In some of those cases the putative primary can be detected by routine IHC stains or at least through gene expression profiling. On the contrary, in some instances morphology, imaging and molecular predictive assays are 'non-contributory': only these samples could be correctly defined as CUPs.

Thus, there are at least two different hypotheses which can be involved in CUP biology: i) the first suggests that CUPs are heterogeneous group of site-specific tumors which share the properties of the small primary from which derive; ii) the second regards to CUP as to a distinct entity with a specific genetic asset. It is clearly evident that the biological and genetic enigma that could be related to CUPs is enclosed in the molecular mechanisms that speed up distant metastatization so that to confer to the primary lesion a status comparable to dormancy. In other words, it is conceivable that CUPs are a distinct biological entity involving specific genetic and phenotypic alterations, although at the present there are no known and validate molecular features to clearly distinguish these cancers. This observation is further supported by a recent epidemiological analysis performed on a CUP cohort from the Swedish Family Cancer Database. This study showed that CUPs cluster with families of kidney, lung, and colorectal cancers and suggested a marked genetic basis in the onset of the syndrome [74].

A wide variety of chromosomal abnormalities have been described in CUPs: aberration of chromosome 1, 6, 7 and 11 are the most frequently reported [75]. Aneuploidy has been seen in 70% of adenocarcinomas of unknown primary: no relationship has been found with the pattern of metastatic spread or the overall survival [76]. Overall data on chromosomal abnormalities can be considered similar to those reported in case of cancers with known primary.

With respect to the oncogenic molecular asset of CUP, several studies have evaluated the expression and the mutational status of both oncogenes and tumor suppressor genes. Unexpectedly--although few studies are available--lesions of the key players know to drive the vast majority of human cancer cannot be documented in CUPs. Expression of c-myc, KRAS, HER2 has been studied by IHC in a series of 26 CUP cases and was present in less than one third of samples [77]. Nevertheless this observation does not allow a clear classification of CUPs. Indeed KRAS alterations, for example, are present in just about 30% of cases of human cancer as a whole and therefore, no significant differences seem to exist between the latter and CUP in this perspective.

Interestingly immunohistochemistry studies demonstrated that EGFR is frequently expressed in CUPs, whereas c-KIT and HER2/neu are infrequently activated. Notably no significant association has been established between EGFR expression level and patients prognosis [78].

The EGFR oncogene seems to be infrequently mutated (1% data by Sanger Institute Catalogue of Somatic Mutations in Cancer--COSMIC--http://www.sanger.ac.uk/cosmic) in CUP whereas the intron 1 cytosine-adenosine (CA) repeat has been reported [79] to be increased in absence of correlation with patients survival and prognosis. Previous observations [80] also demonstrate that p53 mutations rarely occur in CUPs, thus suggesting a minor role of the protein in CUP onset.

Precocious undifferentiated neoplastic spread is the hallmark of CUP and this fact represents a strong rationale to investigate molecular pathways involved in metastatic process. Very recently Koo JS et al. [81] showed that hypoxia-related proteins are expressed in nodal squamous cell metastases of head and neck of unknown primary. The Glut-1, HIF1α, and COX2 expression level seems to be related to a worse patient prognosis.

Metastasis follows the inappropriate activation of a genetic program termed 'invasive growth' (or epithelial-mesenchymal transition), which is a physiological process that occurs during embryonic development and post-natal organ regeneration [82]. Burgeoning evidence indicates that invasive growth is executed by stem and progenitor cells, and is usurped by cancer stem cells. The MET proto-oncogene, which is expressed in both stem and cancer cells, is a key regulator of invasive growth [83]. MET encodes the tyrosine-kinase receptor for "Scatter Factor", a sensor of adverse microenvironmental conditions (such as hypoxia [84] and ionizing radiations [85]) and drives cell invasion and metastasis through the transcriptional activation of the 'invasive growth signature', a genetic program including cell scattering, invasion, protection from apoptosis and angiogenesis [86]. In human cancers, MET activation generally occurs as a late event, mainly consequent to receptor overexpression or gene amplification. Somatic point mutations are rarely found, accounting for no more than 3-4% of unselected primary cancers (data from by Sanger Institute Catalogue of Somatic Mutations in Cancer--COSMIC--http://www.sanger.ac.uk/cosmic).

Several strategies to block the activation of MET are under development, such as the use of tyrosine kinase inhibitors or monoclonal antibodies and some of these compounds have already been used in clinical trials [87].

We have recently demonstrated [88], by a screening of about 50 CUP patients for occurrence of MET somatic mutations, an extremely high MET mutational incidence (about 15%, *vs*. the 1-3% of the general cancer population), in the absence of high mutational background. Nucleotide changes found clustered either in the kinase domain or in the extracellular semaphorin domain. Mutated receptors were functional and sustained the transformed phenotype, suggesting that MET activating mutations are genetic markers associated with the CUP syndrome. Therefore, MET mutation may as well reflect either differentiation grade and/or organ of origin. In this respect a preferential expression of MET in cancer stem cells has been postulated [83].

## Conclusions

Metastases are defined as macroscopic lesions which develop far, through blood or lymphatic vessels from the primary tumor: they are generally resistant to therapy and eventually lead patients to death. The molecular and cellular mechanisms which drive the metastatic spread are the topic of constant debate and scientific research due to the potential implications for cancer patients' prognosis. The process of metastases might begin before the growth of the primary mass. Recent evidence suggests that tumor cells might start conditioning for distant tissues colonization through the establishment of a so called 'pro-metastatic' niche [89]. Early metastatic dissemination is reflected in the clinical absence of symptoms (dormancy) of the primary tumor. CUPs identify a very aggressive pathology which--at the present- is still lacking for appropriate therapies. Indeed these tragic cases may be described as not only of unknown origin with respect to the organ site but also as of unknown pathogenesis. Despite many studies are focused on tracking their putative origin, the real enigma represented by CUPs is related to their own biological and genetic setting. Besides, growing evidence sustains that rationale for personalized targeted therapies is inside the tumors' genome rather than in their tissue of origin. Certainly this is true for all advanced cancers and not specifically for CUPs.

This ideal of personalized oncology has not been reached for most patients and the clinical reality at this time is that at least some CUP patients may be treated with more effective by recognizing their tissue of origin. Patients with advanced cancer whether the primary site is known or not, may eventually be treated based upon specific genomic abnormalities that are discovered in their cancer cells. This is already occurring in some instances. However, knowing the tissue of origin remains an immediate important factor in determining therapy for most patients with advanced cancer.

Nevertheless, further studies should be addressed to deeper investigate the molecular profile of CUPs. Preliminary results suggest a role for MET somatic mutations as driving force in CUP syndrome and put MET among the most promising therapeutic targets. Main address should be also focused on the potential role of the cancer stem cells compartment. Data should be collected to characterize the role played by undifferentiated cancer cells in tumor dissemination and their interaction with surrounding stroma to define molecular markers able to detect cancers displaying occult disseminative potential. This approach will lead to the creation of a new sensitive platform that might select an otherwise heterogeneous group of patients, such as that of patients presenting with metastatic cancers of unknown primary site of origin.

## Abbreviations

CUP: Cancer of unknown primary origin; IHC: Immunohistochemistry; CT: Computed tomography; MRI: Magnetic resonance imaging; PET: Positron emission tomography; FDG: Fluorodeoxy-glucose; ToO: Tissue of origin; RT-PCR: Reverse transcriptase polymerase chain reaction; RNA: Ribonucleic acid; FFPE: Formalin-fixed paraffin-embedded; CK: Cytokeratin; TTF: Thyroid transcription factor; ER: Estrogen receptor; CA125: Cancer antigen 125; VIM: Vimentin; PSA: Prostate specific antigen; CCP-CUP: Colon cancer profile--cancer of unknown primary origin; IMRT: Intensity modulated radiation therapy; K-RAS: v-Ki-ras2 kirsten rat sarcoma viral oncogene homolog; EGFR: Epidermal growth factor receptor; HIF: Hypoxia inducible factor; COX: Cyclooxygenase; GluT: Glucose transporter

## Competing interests

The authors declare that they have no competing interests.

## Authors' contributions

GMS, RS and PC were involved in the conception of the manuscript; AC and MR contribute to collect and analyze bibliographic references; GMS, RS and PC structured and wrote the manuscript. All the authors approved the final version of the manuscript.

## References

[B1] Fizazi K Greco FA Pavlidis N Pentheroudakis G On behalf of the ESMO guidelines working group. Cancers of unknown primary site: ESMO Clinical Practice Guidelines for diagnosis, treatment and follow-up Ann Oncol 2011 22 suppl 6 vi64 vi68 21908507 10.1093/annonc/mdr389

[B2] Abbruzzese JL Abbruzzese MC Hess KR Raber MN Lenzi R Frost P Unknown primary carcinoma: natural history and prognostic factors in 657 consecutive patients J Clin Oncol 1994 12 8 1272 1280 8201389 10.1200/JCO.1994.12.6.1272

[B3] Pavilidis N Fizazi K Carcinoma of unknown primary (CUP) Crit Rev Oncol Hematol 2009 69 271 278 10.1016/j.critrevonc.2008.09.005 18977667

[B4] van de Wouw AJ Jansen RL Speel EJ Hillen HF The unknown biology of the unknown primary tumors: a literature review Annals of Oncology 2003 14 2 191 96 10.1093/annonc/mdg068 12562643

[B5] Holmes FF Fouts TL Metastatic cancer of unknown primary site Cancer 1970 26 4 816 820 10.1002/1097-0142(197010)26:4<816::AID-CNCR2820260413>3.0.CO;2-R 5506606

[B6] Briasoulis E Pavlidis N Cancer of unknown primary origin Oncologist 1997 2 3 142 152 10388044

[B7] Pavlidis N Briasoulis E Hainsworth J Greco FA Diagnostic and therapeutic management of cancer of an unknown primary Eur J Cancer 2003 39 1990 2005 10.1016/S0959-8049(03)00547-1 12957453

[B8] Shu X Liu H Ji J Sundquist K Försti A Sundquist J Hemminki K Subsequent cancers in patients diagnosed with cancer of unknown primary (CUP): etiological insights? Ann Oncol 2011 in press 10.1093/annonc/mdr059 21450937

[B9] Hainsworth JD Gereco FA Treatment of patients with cancer of unknown primary site N Engl J Med 1993 329 4 257 263 10.1056/NEJM199307223290407 8316270

[B10] Mierke CT Cancer cells regulate biomechanical properties of human microvascular endothelial cells J Biol Chem 2011 286 40025 40037 10.1074/jbc.M111.256172 21940631 PMC3220522

[B11] Ecles SA Welch DR Metastases: recent discoveries and novel treatment strategies Lancet 2007 307 9574 1742 1757 10.1016/S0140-6736(07)60781-8 PMC2214903 17512859

[B12] Marchesi F Locatelli M Solinas G Erreni M Allavena P Mantovani A Role of CX3CR1/CX3CL1 axis in primary and secondary involvement of the nervous system by cancer J Neuroimmunol 2010 224 1-2 39 44 10.1016/j.jneuroim.2010.05.007 20630606

[B13] Rowe RG Weiss SJ Breaching the basement membrane: who, when and how? Trends Cell Biol 2008 18 11 560 574 10.1016/j.tcb.2008.08.007 18848450

[B14] Greco FA Oien K Erlander M Osborne R Varadhachary G Bridgewater J Cohen D Wasan H Cancer of unknown primary: progress in the search for improved and rapid diagnosis leading toward superior patient outcomes Ann Oncol 2011 in press 10.1093/annonc/mdr306 21709138

[B15] Dennis JL Hvidsten TR Wit EC Komorowski J Bell AK Downie I Mooney J Verbeke C Bellamy C Keith WN Oien KA Markers of adenocarcinoma characteristic of the site of origin: development of diagnostic algorithm Clin Cancer Res 2005 11 10 3766 3772 10.1158/1078-0432.CCR-04-2236 15897574

[B16] Rossi G Nannini N Costantini M Morphology and more specific immunohistochemical stains are fundamental prerequisites in detection of unknown primary cancer J Clin Oncol 2009 27 4 649 650 19114684 10.1200/JCO.2008.20.3604

[B17] De Jong D Horlings H Wesseling J van't Veer L In reply to Rossi G et al J Clin Oncol 2009 27 4 651 652

[B18] Horlings HM van Laar R Kerst JM Helgason HH Wesseling J van der Hoeven JJ Warmoes MO Floore A Witteveen A Lahti-Domenici J Glas AM Van't Veer LJ de Jong D Gene expression profiling to identify the histogenic origin of metastatic adenocarcinomas of unknown primary J Clin Oncol 2008 26 27 4435 4441 10.1200/JCO.2007.14.6969 18802156

[B19] Varadhachary GR Raber MN Matamoros A Abbruzzese JL Carcinoma of unknown primary with a colon-cancer profile-changing paradigm and emerging definitions Lancet Oncol 2008 9 6 596 10 10.1016/S1470-2045(08)70151-7 18510991

[B20] Greco FA Hainsworth JD DeVita TV, Hellman S, Rosenberg SA Cancer of unknown primary Cancer: Principles and Practice of Oncology 2011 9 Philadelphia: Wolters, Kluwer/Lippincott, Williams and Wilkins 2033 2051

[B21] Pavlidis N Forty years experience of treating cancer of unknown primary Acta Oncol 2007 46 5 592 601 10.1080/02841860701243095 17562435

[B22] Boellaard R Doherty MJ Weber WA Mottaghy FM Lonsdale MN Stroobants SG Oyen WJ Kotzerke J Hoekstra OS Pruim J Marsden PK Tatsch K Hoekstra CJ Visser EP Arends B Verzijlbergen FJ Zijlstra JM Comans EF Lammertsma AA Paans AM Willemsen AT Beyer T Bockisch A Schaefer-Prokop C Delbeke D Baum RP Chiti A Krause BJ FDG PET and PET/CT: EANM procedure guidelines for tumour PET imaging: version 1.0 Eur J Nucl Med Mol Imaging 2010 37 1 181 200 10.1007/s00259-009-1297-4 19915839 PMC2791475

[B23] von Schulthess GK Steinert HC Hany TF Integrated PET/CT: current applications and future directions Radiology 2006 238 2 405 422 10.1148/radiol.2382041977 16436809

[B24] Kwee TC Kwee RM Combined FDG-PET/CT for the detection of unknown primary tumors: systematic review and metaanalysis Eur Radiol 2009 19 3 731 744 10.1007/s00330-008-1194-4 18925401 PMC2816234

[B25] Werner MK Pfannenberg C Öksüz MÖ Nonspecific FDG uptake in the tongue mimicking the primary tumor in a patient with cancer of unknown primary Clin Imaging 2011 35 5 405 407 10.1016/j.clinimag.2010.09.005 21872134

[B26] Dandekar MR Kannan S Rangarajan V Purandare NC Chaukar DA Deshmukh A D'cruz AK Utility of PET in unknown primary with cervical metastasis: a retrospective study Indian J Cancer 2011 48 2 181 186 10.4103/0019-509X.82882 21768663

[B27] Tos T Klyver H Drzewiecki KT Extensive screening for primary tumor is redundant in melanoma of unknown primary J Surg Oncol 2011 in press 10.1002/jso.21994 21721009

[B28] Deron PB Bonte KM Vermeersch HF Van de Wiele C Lymph node metastasis of squamous cell carcinoma from an unknown primary in the upper and middle neck: impact of (18)f-fluorodeoxyglucose positron emission tomography/computed tomography Cancer Biother Radiopharm 2011 6 3 331 4 10.1089/cbr.2010.0918 21711095

[B29] Rudmik L Lau HY Matthews TW Bosch JD Kloiber R Molnar CP Dort JC Clinical utility of PET/CT in the evaluation of head and neck squamous cell carcinoma with an unknown primary: A prospective clinical trial Head Neck 2011 33 7 935 940 10.1002/hed.21566 21674668

[B30] Hu M Zhao W Zhang PL Ju GF Fu Z Zhang GL Kong L Yang YQ Ma YD Yu JM Clinical applications of 18 F-fluorodeoxyglucose positron emission tomography/computed tomography in carcinoma of unknown primary Chin Med J (Engl) 2011 124 7 1010 14 21542959

[B31] Park JS Yim JJ Kang WJ Chung JK Yoo CG Kim YW Han SK Shim YS Lee SM Detection of primary sites in unknown primary tumors using FDG-PET or FDG-PET/CT BMC Res Notes 2011 4 56 10.1186/1756-0500-4-56 21385465 PMC3068107

[B32] Pak K Kim SJ Kim IJ Nam HY Kim BS Kim K Kim YK Clinical implication of (18)F-FDG PET/CT in carcinoma of unknown primary Neoplasma 2011 58 2 135 139 10.4149/neo_2011_02_135 21275463

[B33] Keller F Psychogios G Linke R Lell M Kuwert T Iro H Zenk J Carcinoma of unknown primary in the head and neck: Comparison between positron emission tomography (PET) and PET/CT Head Neck 2010 in press 10.1002/hed.21635 21990221

[B34] Yabuki K Tsukuda M Horiuchi C Taguchi T Nishimura G Role of 18 F-FDG PET in detecting primary site in the patient with primary unknown carcinoma Eur Arch Otorhinolaryngol 2010 267 11 1785 1792 10.1007/s00405-010-1371-3 20814690

[B35] Salem S Patel NH Barwick T Al-Nahhas A Howard DJ Zerizer I Win Z Occult squamous cell carcinoma of the uvula detected by F-18 FDG PET/CT in a case of carcinoma of unknown primary in the head and neck Clin Nucl Med 2010 35 10 800 801 10.1097/RLU.0b013e3181ef09cb 20838291

[B36] Prasad V Ambrosini V Hommann M Hoersch D Fanti S Baum RP Detection of unknown primary neuroendocrine tumours (CUP-NET) using (68)Ga-DOTA-NOC receptor PET/CT Eur J Nucl Med Mol Imaging 2010 37 1 67 77 10.1007/s00259-009-1205-y 19618183

[B37] Roh JL Kim JS Lee JH Cho KJ Choi SH Nam SY Kim SY Utility of combined (18)F-fluorodeoxyglucose-positron emission tomography and computed tomography in patients with cervical metastases from unknown primary tumors Oral Oncol 2009 45 3 218 224 10.1016/j.oraloncology.2008.05.010 18804404

[B38] Cianchetti M Mancuso AA Amdur RJ Werning JW Kirwan J Morris CG Mendenhall WM Diagnostic evaluation of squamous cell carcinoma metastatic to cervical lymph nodes from an unknown head and neck primary site Laryngoscope 2009 119 12 2348 2354 10.1002/lary.20638 19718744

[B39] Padovani D Aimoni C Zucchetta P Paluzzi A Pastore A 18-FDG PET in the diagnosis of laterocervical metastases from occult carcinoma Eur Arch Otorhinolaryngol 2009 266 2 267 271 10.1007/s00405-008-0733-6 18587594

[B40] Kaya AO Coskun U Unlu M Akdemir UO Ozdemir NY Zengin N Benekli M Yildiz R Yaman E Ozturk B Gumus M Uner A Yamac D Ucgul E Buyukberber S Whole body 18 F-FDG PET/CT imaging in the detection of primary tumours in patients with a metastatic carcinoma of unknown origin Asian Pac J Cancer Prev 2008 9 4 683 636 19256759

[B41] Hu M Li MH Kong L Liu NB Yang GR Yu JM (18)F-FDG PET-CT in detecting the primary tumor in patients with metastatic cancers of unknown primary origin Zhonghua Zhong Liu Za Zhi 2008 30 9 699 701 19173915

[B42] Dong MJ Zhao K Lin XT Zhao J Ruan LX Liu ZF Role of fluorodeoxyglucose-PET versus fluorodeoxyglucose-PET/computed tomography in detection of unknown primary tumor: a meta-analysis of the literature Nucl Med Commun 2008 29 9 791 802 18677207 10.1097/MNM.0b013e328302cd26

[B43] Johansen J Buus S Loft A Keiding S Overgaard M Hansen HS Grau C Bundgaard T Kirkegaard J Overgaard J Prospective study of 18FDG-PET in the detection and management of patients with lymph node metastases to the neck from an unknown primary tumor. Results from the DAHANCA-13 study Head Neck 2008 30 4 471 478 10.1002/hed.20734 18023031

[B44] Nassenstein K Veit-Haibach P Stergar H Gutzeit A Freudenberg L Kuehl H Fischer M Barkhausen J Bockisch A Antoch G Cervical Lymph Node Metastases of Unknown Origin: Primary Tumor Detection with Whole-Body Positron Emission Tomography/Computed Tomography Acta Radiol 2007 23 1 8 10.1080/02841850701581768 17963088

[B45] Garin E Prigent-Lejeune F Lesimple T Barge ML Rousseau C Devillers A Bouriel C Habiba MT Bernard AM Bridji B Resche I Impact of PET-FDG in the diagnosis and therapeutic care of patients presenting with metastases of unknown primary Cancer Invest 2007 25 4 232 239 10.1080/07357900701206331 17612933

[B46] Paul SA Stoeckli SJ von Schulthess GK Goerres GW FDG PET and PET/CT for the detection of the primary tumour in patients with cervical non-squamous cell carcinoma metastasis of an unknown primary Eur Arch Otorhinolaryngol 2007 264 2 189 195 17177026 10.1007/s00405-006-0177-9

[B47] Ambrosini V Nanni C Rubello D Moretti A Battista G Castellucci P Farsad M Rampin L Fiorentini G Franchi R Canini R Fanti S 18 F-FDG PET/CT in the assessment of carcinoma of unknown primary origin Radiol Med 2006 111 8 1146 1155 10.1007/s11547-006-0112-6 17171520

[B48] Talantov D Baden J Jatkoe T Hahn K Yu J Rajpurohit Y Jiang Y Choi C Ross JS Atkins D Wang Y Mazumder A A quantitative reverse transcriptase-polymerase chain reaction assay to identify metastatic carcinoma tissue of origin J Mol Diagn 2006 8 3 320 329 10.2353/jmoldx.2006.050136 16825504 PMC1867609

[B49] Varadhachary GR Talantov D Raber MN Meng C Hess KR Jatkoe T Lenzi R Spigel DR Wang Y Greco FA Abbruzzese JL Hainsworth JD Molecular profiling of carcinoma of unknown primary and correlation with clinical evaluation J Clin Oncol 2008 26 27 4442 4448 10.1200/JCO.2007.14.4378 18802157

[B50] Ma XJ Patel R Wang X Salunga R Murage J Desai R Tuggle JT Wang W Chu S Stecker K Raja R Robin H Moore M Baunoch D Sgroi D Erlander M Molecular classification of human cancers using a 92-gene real-time quantitative polymerase chain reaction assay Arch Pathol Lab Med 2006 130 4 465 473 16594740 10.5858/2006-130-465-MCOHCU

[B51] Tothill RW Kowalczyk A Rischin D Bousioutas A Haviv I van Laar RK Waring PM Zalcberg J Ward R Biankin AV Sutherland RL Henshall SM Fong K Pollack JR Bowtell DD Holloway AJ An expression-based site of origin diagnostic method designed for clinical application to cancer of unknown origin Cancer Res 2005 65 10 4031 4040 10.1158/0008-5472.CAN-04-3617 15899792

[B52] Rosenfeld N Aharonov R Meiri E Rosenwald S Spector Y Zepeniuk M Benjamin H Shabes N Tabak S Levy A Lebanony D Goren Y Silberschein E Targan N Ben-Ari A Gilad S Sion-Vardy N Tobar A Feinmesser M Kharenko O Nativ O Nass D Perelman M Yosepovich A Shalmon B Polak-Charcon S Fridman E Avniel A Bentwich I Bentwich Z Cohen D Chajut A Barshack I MicroRNAs accurately identify cancer tissue origin Nat Biotechnol 2008 26 4 462 469 10.1038/nbt1392 18362881

[B53] Varadhachary GR Spector Y Abbruzzese JL Rosenwald S Wang H Aharonov R Carlson HR Cohen D Karanth S Macinskas J Lenzi R Chajut A Edmonston TB Raber MN Prospective gene signature study using microRNA to identify the tissue of origin in patients with carcinoma of unknown primary Clin Cancer Res 2011 17 12 4063 4070 10.1158/1078-0432.CCR-10-2599 21531815

[B54] Ferracin M Pedriali M Veronese A Zagatti B Gafà R Magri E Lunardi M Munerato G Querzoli G Maestri I Ulazzi L Nenci I Croce CM Lanza G Querzoli P Negrini M MicroRNA profiling for the identification of cancers with unknown primary tissue-of-origin J Pathol 2011 225 1 43 53 10.1002/path.2915 21630269 PMC4325368

[B55] Stancel GA Coffey D Alvarez K Halks-Miller M Lal A Mody D Koen T Fairley T Monzon FA Identification of tissue of origin in body fluid specimens using a gene expression microarray assay Cancer Cytopathol 2011 in press 10.1002/cncy.20167 21717591

[B56] Aizenberg MR Fox BD Suki D McCutcheon IE Rao G Rhines LD Surgical management of unknown primary tumors metastatic to the spine J Neurosurg Spine 2011 in press 10.3171/2011.9.SPINE11422 21981272

[B57] Culine S Kramar A Saghatchian M Bugat R Lesimple T Lortholary A Merrouche Y Laplanche A Fizazi K French Study Group on Carcinomas of Unknown Primary Development and validation of a prognostic model to predict the length of survival in patients with carcinomas of an unknown primary site J Clin Oncol 2002 20 24 4679 4683 10.1200/JCO.2002.04.019 12488413

[B58] van de Wouw AJ Janssen-Heijnen ML Coebergh JW Hillen HF Epidemiology of unknown primary tumours; incidence and population based survival of 1285 patients in Southeast Netherlands, 1984-1992 Eur J Cancer 2002 38 409 413 10.1016/S0959-8049(01)00378-1 11818207

[B59] Massard C Loriot Y Fizazi K Carcinomas of an unknown primary origin-diagnosis and treatment Nat Rev Clin Oncol 2011 8 12 701 710 10.1038/nrclinonc.2011.158 22048624

[B60] Golfinopoulos V Pentheroudakis G Salanti G Nearchou AD Ioannidis JP Pavlidis N Comparative survival with diverse chemotherapy regimens for cancer of unknown primary site: multiple-treatments meta-analysis Cancer Treat Rev 2009 35 570 573 10.1016/j.ctrv.2009.05.005 19539430

[B61] Briasoulis E Kalofonos H Bafaloukos D Samantas E Fountzilas G Xiros N Skarlos D Christodoulou C Kosmidis P Pavlidis N Carboplatin plus paclitaxel in unknown primary carcinoma: a phase II Hellenic Cooperative Oncology Group Study J Clin Oncol 2000 18 17 3101 3107 10963638 10.1200/JCO.2000.18.17.3101

[B62] Park YH Ryoo BY Choi SJ Yang SH Kim HT A phase II study of paclitaxel plus cisplatin chemotherapy in an unfavourable group of patients with cancer of unknown primary site Jpn J Clin Oncol 2004 34 11 681 685 10.1093/jjco/hyh124 15613558

[B63] Pentheroudakis G Briasoulis E Kalofonos HP Fountzilas G Economopoulos T Samelis G Koutras A Karina M Xiros N Samantas E Bamias A Pavlidis N Hellenic Cooperative Oncology Group Docetaxel and carboplatin combination chemotherapy as outpatient palliative therapy in carcinoma of unknown primary: a multicentre Hellenic Cooperative Oncology Group phase II study Acta Oncol 2008 47 6 1148 1155 10.1080/02841860701843043 18607872

[B64] Greco FA Burris HA Litchy S Barton JH Bradof JE Richards P Scullin DC Jr Erland JB Morrissey LH Hainsworth JD Gemcitabine, carboplatin, and paclitaxel for patients with carcinoma of unknown primary site: a Minnie Pearl Cancer Research Network study J Clin Oncol 2002 20 6 1651 1656 10.1200/JCO.20.6.1651 11896116

[B65] Hainsworth JD Erland JB Kalman LA Schreeder MT Greco FA Carcinoma of unknown primary site: treatment with 1-hour paclitaxel, carboplatin, and extended-schedule etoposide J Clin Oncol 1997 15 6 2385 2393 9196154 10.1200/JCO.1997.15.6.2385

[B66] Hainsworth JD Spigel DR Thompson DS Murphy PB Lane CM Waterhouse DM Naot Y Greco FA Paclitaxel/carboplatin plus bevacizumab/erlotinib in the first-line treatment of patients with carcinoma of unknown primary site Oncologist 2009 14 12 1189 9 10.1634/theoncologist.2009-0112 19965914

[B67] Krämer A Hübner G Schneeweiss A Folprecht G Neben K Carcinoma of Unknown Primary--an Orphan Disease? Breast Care (Basel) 2008 3 3 164 170 10.1159/000136001 PMC2931112 20824034

[B68] Møller AK Pedersen KD Abildgaard J Petersen BL Daugaard G Capecitabine and oxaliplatin as second-line treatment in patients with carcinoma of unknown primary site Acta Oncol 2010 49 4 431 435 10.3109/02841861003649240 20235750

[B69] Hainsworth JD Spigel DR Burris HA Shipley D Farley C Macias-Perez IM Barton J Greco FA Oxaliplatin and capecitabine in the treatment of patients with recurrent or refractory carcinoma of unknown primary site: a phase 2 trial of the Sarah Cannon Oncology Research Consortium Cancer 2010 116 10 2448 2454 20209610 10.1002/cncr.25029

[B70] Cerezo L raboso E Ballestreros AI Unknown primary cancer of the head and neck: a multidisplinary approach Clin Transl Oncol 2011 13 2 88 97 10.1007/s12094-011-0624-y 21324796

[B71] Frank SJ Rosenthal DI Petsuksiri J Ang KK Morrison WH Weber RS Glisson BS Chao KS Schwartz DL Chronowski GM El-Naggar AK Garden AS Intensity-modulated radiotherapy for cervical node squamous cell carcinoma metastases from unknown head-and-neck primary site: M. D. Anderson Cancer Center outcomes and patterns of failure Int J Radiat Oncol Biol Phys 2010 78 4 1005 1010 10.1016/j.ijrobp.2009.09.006 20207504

[B72] Shoushtari A Saylor D Kerr KL Sheng K Thomas C Jameson M Reibel J Shonka D Levine P Read P Outcomes of Patients with Head-and-Neck Cancer of Unknown Primary Origin Treated with Intensity-Modulated Radiotherapy Int J Radiat Oncol Biol Phys 2011 in press 10.1016/j.ijrobp.2011.01.014 21377283

[B73] Colletier PJ Garden AS Morrison WH Goepfert H Geara F Ang KK Postoperative radiation for squamous cell carcinoma metastatic to cervical lymph nodes from an unknown primary site: outcomes and patterns of failure Head Neck 1998 20 8 674 681 10.1002/(SICI)1097-0347(199812)20:8<674::AID-HED3>3.0.CO;2-H 9790287

[B74] Hemminki K Ji J Sundquist J Shu X Familial risks in cancer of unknown primary: tracking the primary sites J Clin Oncol 2011 29 4 435 440 10.1200/JCO.2010.31.5614 21189391

[B75] Natoli C Ramazzotti V Nappi O Giacomini P Palmeri S Salvatore M Landriscina M Zilli M Natali PG Tinari N Iacobelli S Unknown primary tumors Biochim Biophys Acta 2011 1816 1 13 24 21371531 10.1016/j.bbcan.2011.02.002

[B76] Hedley DW Leary JA Kirsten F Metastatic adenocarcinoma of unknown primary site: abnormalities of cellular DNA content and survival Eur J Cancer Clin Oncol 1985 21 2 185 189 10.1016/0277-5379(85)90171-3 3987755

[B77] Pavlidis N Briassoulis E Bai M Fountzilas G Agnantis N Overexpression of C-myc, Ras and C-erbB-2 oncoproteins in carcinoma of unknown primary origin Anticancer Res 1995 15 6B 2563 2567 8669824

[B78] Massard C Voigt JJ Laplanche A Culine S Lortholary A Bugat R Theodore C Priou F Kaminsky MC Lesimple T Pivot X Coudert B Douillard JY Merrouche Y Fizazi K Carcinoma of an unknown primary: are EGF receptor, Her-2/neu, and c-Kit tyrosine kinases potential targets for therapy? Br J Cancer 2007 97 7 857 861 17876336 10.1038/sj.bjc.6603942 PMC2360401

[B79] Dova L Pentheroudakis G Georgiou I Malamou-Mitsi V Vartholomatos G Fountzilas G Kolaitis N Kitsiou E Pavlidis N Global profiling of EGFR gene mutation, amplification, regulation and tissue protein expression in unknown primary carcinomas: to target or not to target? Clin Exp Metastasis 2007 24 2 79 86 10.1007/s10585-007-9055-0 17390112

[B80] Bar-Eli M Abbruzzese JL Lee-Jackson Frost P p53 gene mutation spectrum in human unknown primary tumors Anticancer Res 1993 13 5A 1619 1623 8239543

[B81] Koo JS Kim H Hypoxia-related protein expression and its clinicopathologic implication in carcinoma of unknown primary Tumour Biol 2011 32 5 893 904 10.1007/s13277-011-0190-5 21598042

[B82] Trusolino L Comoglio PM Scatter factor and semaphorin receptors: cell signalling for invasive growth Nat Rev Cancer 2002 2 4 289 300 10.1038/nrc779 12001990

[B83] Boccaccio C Comoglio PM Invasive growth: a MET-driven genetic programme for cancer and stem cells Nat Rev Cancer 2006 6 8 637 645 10.1038/nrc1912 16862193

[B84] Pennacchietti S Michieli P Galluzzo M Mazzone M Giordano S Comoglio PM Hypoxia promotes invasive growth by transcriptional activation of the met protooncogene Cancer Cell 2003 3 4 347 361 10.1016/S1535-6108(03)00085-0 12726861

[B85] De Bacco F Luraghi P Medico E Reato G Girolami F Perera T Gabriele P Comoglio PM Boccaccio C Induction of MET by ionizing radiation and its role in radioresistance and invasive growth of cancer J Natl Cancer Inst 2011 103 8 645 661 10.1093/jnci/djr093 21464397

[B86] Trusolino L Bertotti A Comoglio PM MET signalling: principles and functions in development, organ regeneration and cancer Nat Rev Mol Cell Biol 2010 11 12 834 848 10.1038/nrm3012 21102609

[B87] Stella GM Benvenuti S Comoglio PM Targeting the MET oncogene in cancer and metastases Expert Opin Investig Drugs 2010 19 11 1381 1394 10.1517/13543784.2010.522988 20868306

[B88] Stella GM Benvenuti S Gramaglia D Scarpa A Tomezzoli A Cassoni P Senetta R Venesio T Pozzi E Bardelli A Comoglio PM MET mutations in cancers of unknown primary origin (CUPs) Hum Mutat 2011 32 1 44 50 10.1002/humu.21374 20949619

[B89] Kaplan RN Riba RD Zacharoulis S Bramley AH Vincent L Costa C MacDonald DD Jin DK Shido K Kerns SA Zhu Z Hicklin D Wu Y Port JL Altorki N Port ER Ruggero D Shmelkov SV Jensen KK Rafii S Lyden D VEGFR1-positive haematopoietic bone marrow progenitors initiate the pre-metastatic niche Nature 2005 438 7069 820 827 10.1038/nature04186 16341007 PMC2945882

